# The relationship between traction spurs, Modic change, vacuum phenomenon, and segmental instability of the lumbar spine

**DOI:** 10.1038/s41598-022-14244-4

**Published:** 2022-06-15

**Authors:** Masatsugu Tsukamoto, Tadatsugu Morimoto, Takaomi Kobayashi, Kazuki Muranaka, Tomohito Yoshihara, Kazumasa Maeda, Motoki Sonohata, Yuichi Kasai, Koji Otani, Masaaki Mawatari

**Affiliations:** 1 Department of Orthopedic Surgery, Faculty of Medicine, Saga University, 5-1-1 Nabeshima, Saga, 849-8501 Japan; 2 Department of Orthopaedics, Faculty of Medicine, Khon Kaen University, Khon Kaen, Thailand; 3 Department of Orthopaedic Surgery, Fukushima Medical University School of Medicine, Fukushima, Japan

**Keywords:** Imaging, Orthopaedics

## Abstract

A thorough understanding of lumbar segmental motion is valuable to treat patients with degenerative lumbar disease, but kinematics associated with indicators of lumbar intervertebral instability [traction spur, Modic changes (MCs) and vacuum phenomenon (VP)] in the lumbar spine have not been well understood. The purpose of this study is to evaluate the relationships between traction spur, MCs and VP to radiographic measurements in the lumbar spine. A total of 525 lumbar discs from L1-2 to L5-S1 of 105 patients were evaluated. The sagittal translation (ST) and sagittal angulation were measured from the radiographs taken flexion–extension. The anterior disc height (ADH) was measured from the lateral radiographs, and ΔADH was measured as the difference from supine to sitting position. Logistic regression analyses were used to detect the association between the existence of traction spur, MCs and VP and related factors. Multivariate analysis showed that the traction spur was significantly related to translational motion (ST > 2 mm, OR 4.74) and the VP was significantly related to vertical motion (ΔADH > 3 mm, OR 1.94). These results suggest that the segments with traction spur and VP should be evaluated carefully because these may be a sign of lumbar intervertebral instability.

## Introduction

Spinal instability in the lumbar spine is considered to be a significant factor in lower back pain and indicates lumbar fusion surgery. Functional flexion–extension radiography is the most widely used method in the imaging diagnosis of lumbar intervertebral instability^1–4^. In addition, magnetic resonance imaging (MRI) has become the "golden standard" in evaluating patients with low back pain. On the other hand, there are several other image findings that have been proposed as indicators of vertebral instability.; traction spur, Modic changes (MCs) and vacuum phenomenon (VP).

Traction spur formation is located 2 or 3 mm from the endplate and has a horizontal orientation. Macnab et al. described traction spur as indicators of intervertebral instability on plane radiographic image and emphasized the characteristics of traction spurs and their associations with unstable lumbar disco-vertebral junctions and excessive or abnormal spinal mobility^5^. Yadav et al. reported on the importance of traction spurs, stating that traction spurs constituted the useful indicators of vertebral segment instability^6^. The intervertebral disc VP refers to the radiographic appearance of fluency caused by the presence of gas, usually in lumbar region; this is one of the characteristics of disc degeneration^7,8^. Liao et al. reported that intradiscal VP should be regarded as a sign of intervertebral instability^9^. MCs are bone marrow and endplate change visible on MRI of patients with degenerative disc disease^10,11^. Hayashi et al. suggests that MCs might play a role in the stability of lumbar spine^12^.

A thorough understanding of lumbar segmental motion is valuable to treat patients with degenerative lumbar disease, but　the relationship between functional radiography and these instability indicators of vertebral instability (traction spur, MCs and VP) is not clear. The current study evaluates the relationship between the segmental instability and degenerative findings detected by radiography, computed tomography (CT) and MRI. The purpose in this study is to determine the relationships between traction spur, MCS, and VP to radiographic measurements in the lumbar spine.

## Materials and methods

Between 2014 and 2016, patients who underwent lumbar spine surgery were evaluated retrospectively. Medical records were reviewed to evaluate clinical characteristics and radiological findings. Radiography, CT and MRI studies performed at our institution were reviewed.

The inclusion criteria were defined as patients who had undergone lumbar spine surgery at a single institution from August 2014 and March 2016. The exclusion criteria were trauma (n = 3), infection (n = 19), spinal tumors (n = 8), history of lumbar surgery (n = 16), osteoporotic vertebral fracture (n = 18), and unsuitable radiographs to measure (n = 15). Of 184 patients registered during the study entry period, 105 patients of them were completed in this study [60 men and 45 women, average age of 68.0 ± 12.8 years (range 29–89)]. The diseases responsible for enrollment of patients in the study included the following: lumber spinal stenosis in 72 cases (68.6%), herniated lumbar disc in 15 cases (14.3%), degenerative spondylolisthesis in 9 cases (8.6%), and others in 9 cases (8.6%). A total of 525 lumbar discs from L1-2 to L5-S1 were evaluated for all patients. The Institutional Review Board of the University of Saga at Saga city approved this study and informed consent was obtained from all participants **(**2020-04-R-10). This study also adhered to the principles of the Declaration of Helsinki. Written informed consent was obtained from all patients.

### Radiographic studies

Radiography, CT, and MRI studies were performed on all patients. Plane CT was evaluated with regard to the presence of osteophyte. An anterior lumbar vertebral osteophyte should be > 2 mm or more in length according to the classification of Macnab et al.^4^ Kasai et al. distributed anterior lumbar vertebral osteophytes into six types based on the direction of extension of each pair of osteophytes across the intervertebral disc space as follows: group A, no osteophytes; group B, the pair of osteophytes extended in the direction of the adjacent disc; group C, there was almost complete bone bridge formation by a pair of osteophytes across the intervertebral disc space; group D, the pair of osteophytes extended in a direction away from the adjacent disc; group E, the osteophytes extended nearly horizontally to the vertebral body border without closing the intervertebral disc space; and group F, ungroupable^13^. In this study, anterior lumbar vertebral osteophytes were distributed into three types based Kasai’s classification: no osteophytes, include group A; claw spur, include group B and C; traction spur, include group D and E. For each level, a diagnosis was made of no osteophyte, claw spur, and traction spur. When two different osteophytes were present in one segment, only one diagnosis was applied (first priority: traction spur; and second priority: claw spur). MCs were classified into none or types 1, 2, and 3, according to their signal patterns on T1- and T2-weighted sagittal MR images^10^. VP were evaluated by presence of areas of gaseous radiolucency using CT imaging. The presence of VP was judged as present or not present.

The lateral radiographs of lumbar spine were taken in lateral recumbent (natural, flexion and extension), supine, and sitting positions, respectively. Radiographic parameters, including anterior disc height (ADH), intervertebral slip angle, and distance of slippage, were collected. We measured these parameters using Virtual Place RAIJIN Ver3.8 (AZE Ltd., Tokyo, Japan).

**Figure 1:**
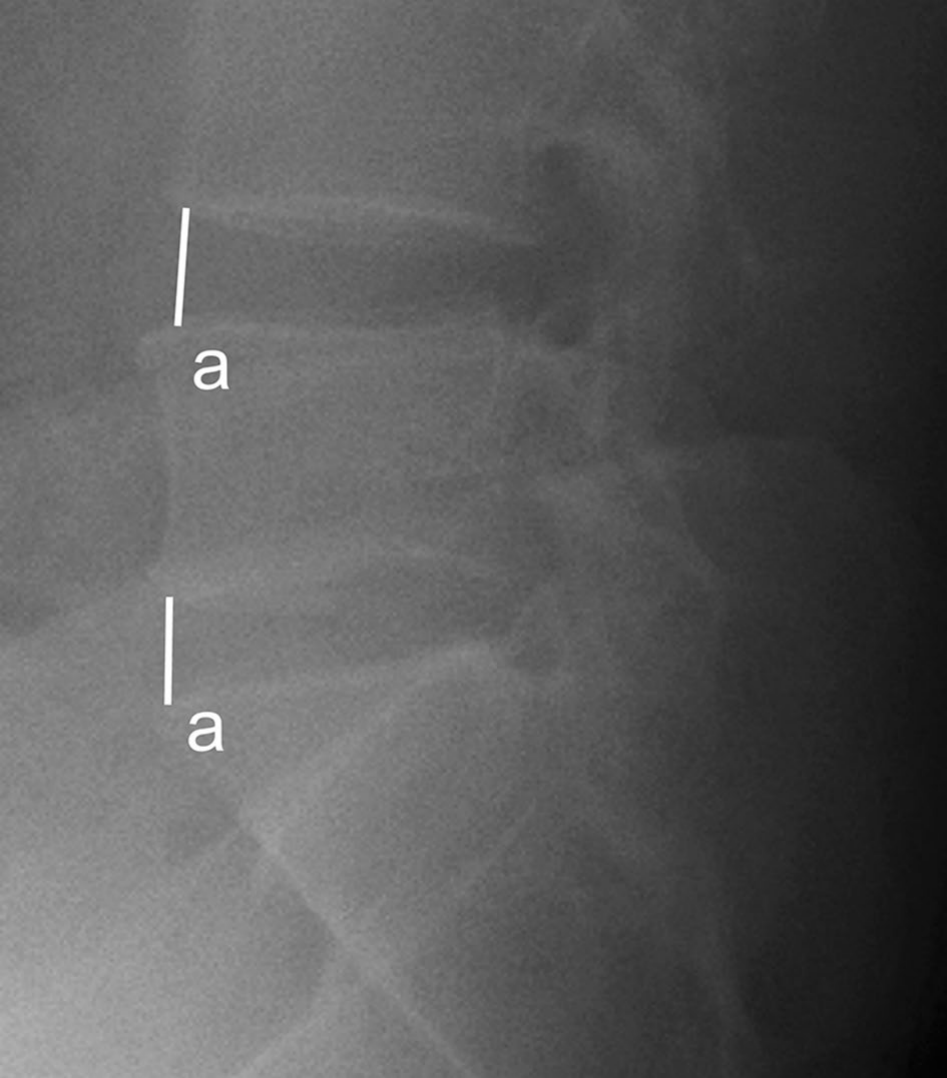
Anterior disc height (ADH): ADH was measured as the distance between the most anterior point of the upper and lower endplates.

ADH was measured as the distance between the most anterior point of the upper and lower endplates. $\Delta$ADH was measured as the difference from supine to sitting position (Fig. 1).

**Figure 2:**
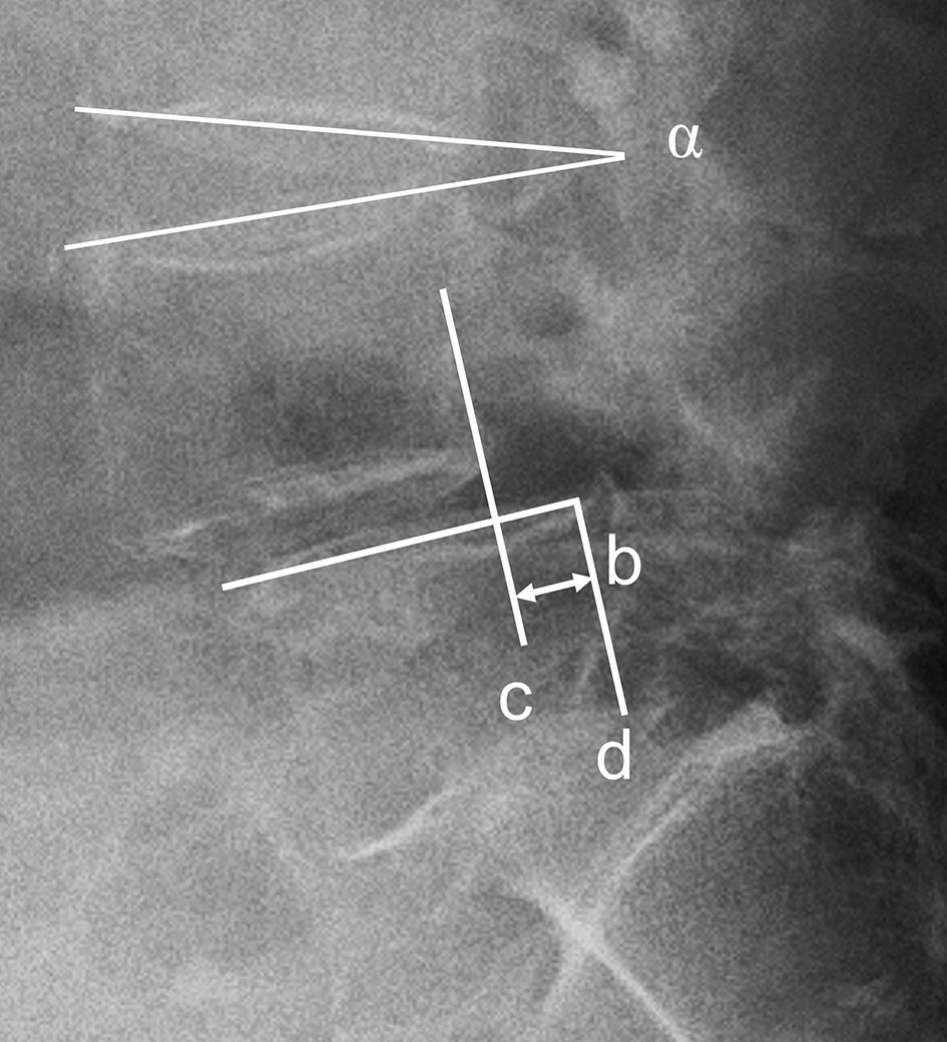
Radiographs of lumbar spine with spondylolisthesis to demonstrate the measurement technique. Sagittal translation (ST): first, the perpendicular distance between parallel lines c and d was measured on radiographs, the distance between these two parallel lines was the distance of slippage (b). The difference of the distance of slippage between flexion and extension was ST. Sagittal angulation (SA): the intervertebral slip angle(α) was the angle between two lines at the endplates of the affected disc. The difference of intervertebral slip angles between flexion and extension radiographs was SA.

The distance of slippage was defined by drawing two lines perpendicularly to the line superior endplate of lower vertebral body. The first line was sited at the posterosuperior corner of the caudal vertebra, and the second line was dropped from the posteroinferior corner of cranial vertebra. The distance between these two parallel lines was the distance of slippage. The amount of sagittal translation (ST) was obtained as the difference of the distance of slippage between flexion and extension (Fig. 2).

The intervertebral slip angle was found simply by constructing two lines at the endplates of the affected disc and measuring the angle between them. The sagittal angulation (SA) was also measured as the difference of intervertebral slip angle from extension to flexion (Fig. 2).

Detailed measurement methods were described previously elsewhere^14^. In this study, excessive motion (instability) was defined as following; $\Delta$ADH > 3 mm, ST > 2 mm and SA > 10 degree^14^. We measured the following characteristics twice; radiographic parameter ($\Delta$ADH, ST and SA), the type of spurs, MCs and VP**.** When there was a difference, the first measurement was used.

### Statistical analysis

All statistical analyses were performed with EZR (Saitama Medical Center, Jichi Medical University, Saitama, Japan), which is a graphical user interface for R (The R Foundation for Statistical Computing, Vienna, Austria). More precisely, it is a modified version of R commander designed to add statistical functions frequently used in biostatistics^15^.

Statistically significant differences in radiographic parameter ($\Delta$ADH, ST and SA) between the type of spurs, MCs were assessed using one-way analysis of variance (ANOVA) followed by post hoc multiple comparisons using the Bonferroni method. T-test was used to evaluate the VP and radiographic parameters. After the variables were categorized, multivariate logistic regression analyses were used to compute odds ratios (ORs) and 95% confidence intervals (CIs) and detect the association between the existence of radiographic parameter ($\Delta$ADH, ST and SA) and spur type, MCs and VP. The variables in the multivariate model were that were sex, age, spur type, MCs and VP. The level of significance (*p* value) was set at 0.05.

Inter-observer agreement between the two board certificated spine surgeons of JSSR (The Japanese Society for Spine Surgery and Related Research Reserved), and intra-observer agreement by one reader were analyzed in 30 discs using kappa statistics. The intra- and inter-observer agreement for the type of spur, MCs and VP were analyzed.

## Results

### Reliability of diagnosis of traction spur, Modic changes and vacuum phenomenon

The kappa values for type of spur were 0.81 (95% CI 0.67–0.94) for inter-observer and 0.87 (95% CI 0.69–1.04) for intra-observer. The kappa values for VP were 0.94 (95% CI 0.88–1.02) for inter-observer and 1 for intra-observer. The kappa values for MCs were 0.78 (95% CI 0.60–0.95) for inter-observer and 0.77 (95% CI 0.46–1.08) for intra-observer. The reliability of parameter measurements and evaluations in this study was confirmed.

### Prevalence of traction spur, Modic changes and vacuum phenomenon

The traction spurs were found 219 discs (41.7%), MCs were found in 106 discs (20.6%) and VP was found in 179/525 discs (35.4%) of the 525 discs analyzed (Table 1). The results of the radiographic measurements are shown in Table 2.

**Table 1:** Demographic data.

	Discs (%)
**Age**	
Ave	67.97 ± 12.81
**Sex**	
Male	300 (57.1)
Female	225 (42.9)
**Spur type**	
Claw	115 (21.9)
Traction	219 (41.7)
No spurs	191 (36.4)
**MCs**	
Type 1	17 (3.3)
Type 2	39 (7.6)
Type 3	50 (9.7)
No MCs	409 (79.4)
**VP**	
Positive	179 (34.1)
Negative	346 (65.9)

MCs, Modic changes; VP, vacuum phenomenon.

**Table 2:** Average values of ΔADH and ST and SA, and the percentage of intervertebral disc defined as excessive motion (instability). The excessive motion (instability) was defined as following; ΔADH > 3 mm, ST > 2 mm and SA > 10 degree.

Radiographic parameter	n (%)
**ΔADH (mm)**	
Ave	2.08 ± 1.88
> 3 mm	122 (23.9)
**ST (mm)**	
Ave	0.45 ± 1.01
> 2 mm	41 (8.8)
**SA (degree)**	
Ave	3.81 ± 4.12
> 10°	32 (6.9)

ADH, anterior disc height; ST, sagittal translation; SA, sagittal angulation.

### Relationship between traction spur, Modic changes, vacuum phenomenon and vertical motion ($\Delta$ADH)

There was significant difference between $\Delta$ADH and spur type. $\Delta$ADH was significantly higher in traction spur than the others (*p* < 0.001). There was significant difference between ADH and MCs. $\Delta$ADH was significantly higher in Modic type 3 than Modic type 0 and type 2 (*p* < 0.001, *p* = 0.036, respectively). $\Delta$ADH was significantly higher in VP (Table 3).

**Table 3:** Comparison between radiographic parameter and spur type, Modic changes, vacuum phenomenon.

	ΔADH (mm)	ST (mm)	SA (degree)
**Spur**			
No	1.9 ± 1.7	0.27 ± 0.74	4.0 ± 4.5
Claw	1.6 ± 1.5	0.29 ± 0.91	4.1 ± 3.7
Traction	2.5 ± 2.2	0.70 ± 1.19	3.5 ± 4.0
(*p* value)	*p* < 0.001	*p* < 0.001	*p* = 0.447
**MCs**			
No	1.9 ± 1.7	0.41 ± 0.98	4.0 ± 4.2
Type 1	2.0 ± 1.5	0.89 ± 1.21	1.8 ± 4.2
Type 2	2.0 ± 1.3	0.64 ± 1.14	3.4 ± 3.2
Type 3	2.5 ± 2.2	0.48 ± 0.89	3.0 ± 3.9
(*p* value)	*p* = 0.0012	*p* = 0.183	*p* = 0.083
**VP**			
No	1.8 ± 1.6	0.40 ± 0.97	4.5 ± 4.1
VP	2.6 ± 2.2	0.56 ± 1.09	2.5 ± 3.9
(*p *value)	*p* < 0.001	*p* = 0.0859	*p* < 0.001

ADH, anterior disc height; ST, sagittal translation; SA, sagittal angulation; MCs, Modic changes; VP, vacuum phenomenon.

Factors related to $\Delta$ADH > 3 mm were evaluated using logistic regression analysis to control confounding factors. Using crude analysis between $\Delta$ADH > 3 mm and < 3 mm, significant differences were found in traction spur (*p* = 0.013) and VP (*p* < 0.001). Next, a multiple logistic regression model was used to adjust for age, sex, spur type, MCs and VP. After adjustment for potential confounding factors, significantly elevated adjusted odds ratio (AOR) was observed in segments with VP (AOR 1.94, 95% CI 1.13–3.33, *p* = 0.0164) (Table 4).

**Table 4:** Associations of traction spur, Modic changes, vacuum phenomenon with prevalence of ΔADH > 3 mm.

Factor	Crude analysis			Adjusted analysis		
	OR	95%CI	p value	AOR	95%CI	p value
Age	1.0	0.984–1.02	0.953	0.996	0.979–1.01	0.679
**Sex (vs male)**						
Female	0.799	0.527–1.21	0.290	0.76	0.489–1.19	0.238
**spur type (vs no)**						
Claw	0.593	0.31–1.13	0.114	0.574	0.283–1.16	0.123
Traction	*1.78*	*1.13–2.81*	*0.013*	1.33	0.767–2.31	0.308
**MCs (vs no)**						
Type 1	2.06	0.728–5.81	0.173	1.15	0.382–3.48	0.801
Type 2	1.02	0.446–2.31	0.97	0.596	0.243–1.46	0.258
Type 3	1.51	0.789–2.9	0.213	0.868	0.424–1.78	0.7
**VP (vs no)**						
Positive	*2.14*	*1.41–3.25*	*0.00035*	*1.94*	*1.13–3.33*	*0.016*

The crude analysis used single-variate logistic regressions whereas the adjusted analysis used multivariate logistic regression including all explanatory variables.

ADH, anterior disc height; MCs, Modic changes; VP, vacuum phenomenon; OR, odds ratio; AOR, adjusted odds ratio.

Significant values are in italics.

### Relationship between traction spur, Modic changes, vacuum phenomenon and sagittal translation (ST)

There was a significant difference between ST and spur type. The ST was significantly higher in traction spur than the others (*p* < 0.001). There was no significant difference between ST and MCs or VP (Table 3).

Using crude analysis between ST > 2 mm and < 2 mm, significant differences were found in spur type (*p* = 0.002) (Table 5). Next, a multiple logistic regression model was used, significantly elevated ORs were observed in segments with traction spur (AOR 4.74, 95% CI 1.79–12.6, *p* = 0.00174) (Table 5).

**Table 5:** Associations of traction spur, Modic changes, vacuum phenomenon with prevalence of ST > 2 mm.

Factor	Crude analysis			Adjusted analysis		
	OR	95% CI	*p* value	AOR	95% CI	*p* value
Age	0.998	0.973–1.02	0.893	0.993	0.965–1.02	0.65
**Sex (vs male)**						
Female	0.827	0.429–1.59	0.570	1.04	0.518–2.11	0.903
**Spur type (vs no)**						
Claw	1.41	0.462–4.33	0.544	1.08	0.288–4.07	0.907
Traction	*3.83*	*1.63–9.02*	*0.002*	*4.74*	*1.79–12.6*	*0.002*
**MCs (vs no)**						
Type 1	2.85	0.766–10.6	0.118	0.616	0.256–1.48	0.279
Type 2	1.9	0.619–5.85	0.262	2.32	0.549–9.81	0.253
Type 3	1.44	0.526–3.93	0.479	1.48	0.421–5.19	0.543
**VP (vs no)**						
Positive	1.38	0.717–2.65	0.337	1.05	0.353–3.14	0.928

The crude analysis used single-variate logistic regressions whereas the adjusted analysis used multivariate logistic regression including all explanatory variables.

ST, sagittal translation; MCs, Modic changes; VP, vacuum phenomenon; OR, odds ratio; AOR, adjusted odds ratio.

Significant values are in italics.

### Relationship between traction spur, Modic changes, vacuum phenomenon and sagittal angulation (SA)

There was no significant difference between SA and spur type, and MCs. The discs with VP had significantly smaller SA than those without VP (Table 3).

Using crude analysis between SA > 10 degree and < 10 degree, significant differences were not found (Table 6). Next, a multiple logistic regression model was used, significantly depressed ORs were observed in segments with VP (AOR 0.174, 95% CI 0.0451–0.672, *p* = 0.011) (Table.6).

**Table 6:** Associations of traction spur, Modic changes, vacuum phenomenon with prevalence of SA > 10 degree.

Factor	Crude analysis			Adjusted analysis		
	OR	95% CI	*p* value	AOR	95% CI	*p* value
Age	0.975	0.95–1	0.893	0.985	0.958–1.01	0.279
**Sex (vs male)**						
Female	0.884	0.426–1.84	0.742	0.792	0.366–1.72	0.555
**Spur type (vs no)**						
Claw	0.563	0.197–1.61	0.285	0.779	0.248–2.45	0.669
Traction	0.785	0.358–1.72	0.545	1.78	0.74–4.3	0.198
**MCs (vs no)**						
Type 1	9.9E−08	0-Infinity	0.992	2.71E−07	0-Infinity	0.992
Type 2	9.9E−08	0-Infinity	0.989	2.52E−07	0-Infinity	0.99
Type 3	0.799	0.233–2.73	0.720	*1.30*	0.341–4.96	0.7
**VP (vs no)**						
Positive	1.38	0.717–2.65	0.337	0.174	0.045–0.672	0.011

The crude analysis used single-variate logistic regressions whereas the adjusted analysis used multivariate logistic regression including all explanatory variables.

SA, sagittal angulation; MCs, Modic changes; VP, vacuum phenomenon; OR, odds ratio; AOR, adjusted odds ratio.

Significant values are in italics.

## Discussion

To evaluate the relationships between traction spur, VP, and MCs to radiographic measurements in the lumbar spine, we performed a cross-sectional retrospective radiographic, CT and MRI study to assess discs (intervertebral space) of consecutive patients who underwent spinal surgery. To the best of our knowledge, this study is the first to evaluate various aspects of indicators of vertebral instability (traction spur, VP and MCs) and radiographic instability in lumbar spine. The results showed that traction spur was significantly related to translational motion (ST > 2 mm), VP were significantly related to vertical motion ($\Delta$ADH > 3 mm), however SA was not significantly related any indicators of vertebral instability (traction spur, VP and MCs). These findings may be helpful in the indications for lumbar fusion in clinical practice.

### The association between traction spurs and radiographic measurements

Our multivariate analysis showed traction spur were significantly related to ST > 2 mm. However, we did not find a significant association between traction spur to $\Delta$ADH > 3 mm and SA. Disc degeneration with disc height narrowing is considered related to spinal instability^16^. On the other hand, when disc height is collapsed, there is a natural tendency to restabilize the motion segment and as a result, spondylolisthesis becomes less likely to progress^17^. Murata et al. found that severe disc degeneration was less significantly related to angular displacement and had a tendency to stabilize the motion segment^18^. The values of 10° for sagittal angulation and 3 or 4 mm for sagittal translation are typically used to infer instability^14,19^. In this study, because traction spur associate with > 2 mm ST, traction spur were suggested to indicate intervertebral instability. We speculate that the significant association at ST > 2 mm, but not > 3 mm or > 4 mm, is because traction spur occurs relatively early in disc degeneration.

### The association between vacuum phenomenon and radiographic measurements

Our multivariate analysis showed VP were significantly related to $\Delta$ADH > 3 mm and SA > 10 degree. Because instability may create excessive intervertebral distraction and subsequent negative intradiscal pressure, allowing interstitial nitrogen in the surrounding tissues to become gaseous and to accumulate within cleft of the degenerated disc, it is assumed that the vacuum phenomenon is often associated with vertebral instability^1^. The changes in disc height between supine and sitting positions probably represent two factors: disc elasticity and instability due to disc degeneration. Because of the few younger patients in this study, intervertebral discs with $\Delta$ADH > 3 mm mm may indicate instability due to disc degeneration rather than elasticity. The VP was suggested to be one of the indicators of disc degeneration and intervertebral instability, since VP was associated with $\Delta$ ADH > 3 mm and SA > 10 degree. Clinically, Liao et al. reported that the vacuum sign at the spondylolisthesis segment should be regarded as another sign of instability and suggested that instrumented posterolateral fusion simultaneously with intervertebral fusion with a cage can overcome this situation^9^. Our results also suggest that VP could be a useful indicator in deciding on the adaptation for spinal instrumentation.

### The association between Modic changes and radiographic measurements

Kirkaldy-Willis and Farfan postulated 3 stages with different conditions of stability and motion in the degenerative lumbar spine: dysfunction, instability, and stabilization^1^. Because instability may create excessive intervertebral distraction and subsequent negative intradiscal pressure, allowing interstitial nitrogen in the surrounding tissues to become gaseous and to accumulate within cleft of the degenerated disc, it is assumed that the vacuum phenomenon is often associated with vertebral instability. Moderate disc degeneration with mild disc space narrowing and osteosclerosis of endplate also have been associated with vertebral instability. MCs are bone marrow and endplate change visible on MRI of patients with degenerative disc disease^10,20^. Some previous studies demonstrated the relationship between fusion surgery and MCs and have reported on the instability related to MCs^21–24^. However our multivariate analysis showed MCs were not significantly related to any radiographic parameters. The results suggested that the changes in the endplate are not directly related to intervertebral instability.

### Limitation

The present study was associated with several limitations. Firstly, the subjects were patients who had undergone preoperative radiographic imaging before elective spinal surgery, and an age-matched general population was not used as a control. Thus, the incidence of traction spurs in our subjects would be much higher in comparison to age-matched individuals in the general population. Secondly, this was a cross-sectional study, which cannot clearly explain the prognosis of developing disc degeneration or spinal instability in the presence of traction spurs at each lumbar spinal segment. We are also planning to investigate the direction of spur formation in a longitudinal study. Another limitation of this study is the relatively small number of lumbar segments with > 2 mm sagittal translation and > 10 degree sagittal angulation, which might have influenced the statistical power. Further studies are needed to increase the number of these cases. Finally, the prevalence of MC type 3 was high in this study. Previous reports have indicated that MC types 1 and 2 are more prevalent^11^. On the other hand, MC type 3 has been considered the final stage of degeneration. In this study, since there were many elderly surgical cases, many of them had advanced intervertebral disc degeneration, which might be the reason why MC type 3 was more prevalent in this study.

## Conclusion

Our multivariate analysis showed traction spurs and VP were significantly related to vertical and translational motion. In addition, clinically, our study suggests that the segments with traction spur and VP should be evaluated carefully because these may be a sign of a disc degeneration and instability. The presence of these should be taken into consideration when evaluating stability in the lumbar spine. Characterizing the type of spur observed may be one of the important factors to take into account when making a decision for or against spinal fusion.

## Data Availability

The datasets generated and/or analyzed during the current study are available from the corresponding author on reasonable request.
